# Supplementary material for: Comparative transcriptome analysis of panicle development under heat stress in two rice (Oryza sativa L.) cultivars differing in heat tolerance
Source: PeerJ. 2019 Aug 29;7:e7595. doi: 10.7717/peerj.7595 (PMC6717657; doi:10.7717/peerj.7595)
Supplement: Table S1 [file peerj-07-7595-s001.docx]

**Supplementary Table S1.** Temperatures in growth chambers

| Period | High-temperature chamber  (°C) | Normal-temperature chamber  (°C) |
| --- | --- | --- |
| 0:00:00 − 6:29:59 | 33 | 25 |
| 6:30:00 − 9:29:59 | 35 | 27 |
| 9:30:00 − 17:29:59 | 40 | 32 |
| 17:30:00 − 22:29:59 | 35 | 27 |
| 22:30:00 − 23:59:59 | 33 | 25 |
