# Supplementary material for: Comparative transcriptome analysis of panicle development under heat stress in two rice (Oryza sativa L.) cultivars differing in heat tolerance
Source: PeerJ. 2019 Aug 29;7:e7595. doi: 10.7717/peerj.7595 (PMC6717657; doi:10.7717/peerj.7595)
Supplement: Table S2 [file peerj-07-7595-s002.docx]

**Table S2. Primers used for quantitative real-time PCR in this study**

| Gene | Forward primer 5' → 3' | Reverse primer 5' → 3' |
| --- | --- | --- |
| OsUBQ | AACCAGCTGAGGCCCAAGA | ACGATTGATTTAACCAGTCCATGA |
| BGIOSGA022020 | GGCCTGGCGTTCGAGTTCCA | TCGCCGAGGAGGAGGATGGT |
| BGIOSGA006348 | TGAGAAGGGAGAAGAGTGC | CCTCAGGAAAGTTGGGTT |
| BGIOSGA017088 | GTCAGCCCGACCAGCAGCAC | GGGGTCGAGGAACAGGAACG |
| BGIOSGA006285 | CATCGTGGCTGCGCTGACG | GCCGACCATCTCCTCCTCCC |
| BGIOSGA024710 | GGAGGAGGGCTGTATGTGAA | TAGGCAATGGCGAACTGG |
| BGIOSGA032653 | ACGCCCTGTTCACCCTCA | TCTGCACCCTTGGATTCCTC |
| BGIOSGA015767 | AGGATTACGCCAAGTTCTACGA | AGACCTCCTTCTGCCCTTC |
| BGIOSGA033867 | ACGTGGCGATGGGGATGA | GCCACCTTGCGGAACAGC |
| BGIOSGA025186 | CGGTCCAGTTTACATCGG | CTTGAACAGTGCGTGCTT |
| BGIOSGA009154 | ACGGCCAGCACCGCATCAGT | GCTCTTCATCACCAGCACCCT |
| BGIOSGA005140 | CAAGGAGCACAGTCCACAA | GAAGATAGGAACCCAACCAG |
| BGIOSGA026140 | TCTACTTCCCATTCACCAA | TTCCGACCATACAACTCAA |
| BGIOSGA026976 | GCCGTCTTGAGTCCATCC | TGAGTTTCGGGTTCCTAT |
| BGIOSGA000509 | CTGCCTGTCCGTCCACTTC | CCTTGCCCTTGTCCCACT |
| BGIOSGA007252 | CACCCAGGACCTCTACGACTC | CGACGTTGCGGTTGAGAA |
| BGIOSGA000905 | CTTGCCCTGTGGTCATAC | CGTTGCTAGTTCCTCGTC |
| BGIOSGA016976 | AATCTGCTCGTTCGTTCC | GTTTGATTTGCCTGGTTG |
| BGIOSGA002743 | ACGAGGTGCAGGTGGTGAGG | TGTCGAGCTTGAGCTTGGTGA |
| BGIOSGA006470 | CCGCCTTCGCAGCTGTGG | CCGTTCCCGCCATCACCG |
| BGIOSGA024861 | CTACAACTCCTACTGGCAAGGG | GCGAAGTGCTAACAGGGTGA |
| BGIOSGA028445 | CTCAAAGGCGACCAATCT | ATCCAGCAGCCCTACAAT |
| BGIOSGA040494 | AGGAGCGAAAGGTGGAGG | GGGCGGAGGAGTTTGATT |
| BGIOSGA015108 | ATGATGCGACGCCGCCTTCT | CGGTCATCGGCACCTTCTCC |
| BGIOSGA033644 | TGTCACGACCATTTGGAG | CCTGGAGGGTCAATTCCT |
| BGIOSGA016149 | GGCGATGAGATTCCTTTA | CCTTCTGGGACATTGTTG |
| BGIOSGA002779 | AACAAGGTTGGAACGGATAA | TTGAATGGCACGCAAGTA |
| BGIOSGA001370 | TAGGGTTGAGCGGCTTTAGG | CGATGCCATCTCGTCTTCCT |
| BGIOSGA028077 | ATGTATGGGATGTTGAAGC | CTGGGAACGAAATGCTCT |
