# Supplementary material for: Comparative transcriptome analysis of panicle development under heat stress in two rice (Oryza sativa L.) cultivars differing in heat tolerance
Source: PeerJ. 2019 Aug 29;7:e7595. doi: 10.7717/peerj.7595 (PMC6717657; doi:10.7717/peerj.7595)
Supplement: Table S3 [file peerj-07-7595-s003.docx]

**Supplementary Table S3. Summary of Illumina transcriptome reads mapped to the reference genome**

| Sample | Reads No. | Clean Reads No. | Total_Mapped | Multiple_Mapped | Uniquely_Mapped | Map_Events | Mapped_to_Gene | Mapped_to_InterGene | Mapped_to_Exon |
| --- | --- | --- | --- | --- | --- | --- | --- | --- | --- |
| HHZ_32-1 | 44880360 | 44674622 | 39414676 | 1309808 | 38104868 | 38104868 | 34191031 | 3913837 | 32825909 |
| HHZ_32-2 | 46847432 | 46628266 | 41037200 | 1445145 | 39592055 | 39592055 | 35587941 | 4004114 | 34235249 |
| HHZ_32-3 | 40967374 | 40795800 | 36051296 | 1239348 | 34811948 | 34811948 | 31257732 | 3554216 | 30066120 |
| HHZ_40-1 | 44620298 | 44364488 | 38321314 | 1420423 | 36900891 | 36900891 | 32874937 | 4025954 | 31296686 |
| HHZ_40-2 | 42561626 | 42335318 | 36679902 | 1231999 | 35447903 | 35447903 | 31568967 | 3878936 | 30058438 |
| HHZ_40-3 | 49357800 | 49070296 | 42445633 | 1517388 | 40928245 | 40928245 | 36447308 | 4480937 | 34695128 |
| IR36_32-1 | 46674760 | 46394628 | 40575194 | 1595533 | 38979661 | 38979661 | 35004444 | 3975217 | 33729896 |
| IR36_32-2 | 40207068 | 39995352 | 35087752 | 1220476 | 33867276 | 33867276 | 30495327 | 3371949 | 29449876 |
| IR36_32-3 | 50751686 | 50352482 | 42962629 | 1448253 | 41514376 | 41514376 | 37347235 | 4167141 | 36023767 |
| IR36_40-1 | 47807328 | 47586770 | 41628830 | 1545892 | 40082938 | 40082938 | 35695015 | 4387923 | 34097125 |
| IR36_40-2 | 51302248 | 51067680 | 44638354 | 1845464 | 42792890 | 42792890 | 38272405 | 4520485 | 36600210 |
| IR36_40-3 | 40285562 | 40104338 | 34987195 | 1291697 | 33695498 | 33695498 | 30067663 | 3627835 | 28728657 |

Note: HHZ_32-1, HHZ_32-2, HHZ_32-3 represented three biological replicates in HHZ under 32°C treatment；HHZ_40-1, HHZ_40-2, HHZ_40-3 represented three biological replicates in HHZ under 40°C treatment；IR36_32-1, IR36_32-2, IR36_32-3 represented three biological replicates in IR36 under 32°C treatment；IR36_40-1, IR36_40-2, IR36_40-3 represented three biological replicates in IR36 under 40°C treatment.
